# A split and rearranged nuclear gene encoding the iron-sulfur subunit of mitochondrial succinate dehydrogenase in Euglenozoa

**DOI:** 10.1186/1756-0500-2-16

**Published:** 2009-02-03

**Authors:** Ryan MR Gawryluk, Michael W Gray

**Affiliations:** 1 Department of Biochemistry and Molecular Biology, Dalhousie University, Halifax, Nova Scotia B3H 1X5, Canada

## Abstract

**Background:**

Analyses based on phylogenetic and ultrastructural data have suggested that euglenids (such as *Euglena gracilis*), trypanosomatids and diplonemids are members of a monophyletic lineage termed Euglenozoa. However, many uncertainties are associated with phylogenetic reconstructions for ancient and rapidly evolving groups; thus, rare genomic characters become increasingly important in reinforcing inferred phylogenetic relationships.

**Findings:**

We discovered that the iron-sulfur subunit (SdhB) of mitochondrial succinate dehydrogenase is encoded by a split and rearranged nuclear gene in *Euglena gracilis *and trypanosomatids, an example of a rare genomic character. The two subgenic modules are transcribed independently and the resulting mRNAs appear to be independently translated, with the two protein products imported into mitochondria, based on the presence of predicted mitochondrial targeting peptides. Although the inferred protein sequences are in general very divergent from those of other organisms, all of the required iron-sulfur cluster-coordinating residues are present. Moreover, the discontinuity in the euglenozoan SdhB sequence occurs between the two domains of a typical, covalently continuous SdhB, consistent with the inference that the euglenozoan 'half' proteins are functional.

**Conclusion:**

The discovery of this unique molecular marker provides evidence for the monophyly of Euglenozoa that is independent of evolutionary models. Our results pose questions about the origin and timing of this novel gene arrangement and the structure and function of euglenozoan SdhB.

## Background

Succinate dehydrogenase (SDH, Complex II) is a membrane-anchored protein complex of the mitochondrial and bacterial electron transport chain that catalyzes the oxidation of succinate to fumarate, and that reduces FAD to FADH_2 _in the process (although it is capable of the reverse reaction under favorable conditions). High-resolution crystal structures of Complex II from bacterial (*E. coli*; [1]), avian (chicken; [2]) and mammalian (pig; [3]) sources demonstrate that it is a heterotetramer consisting of the succinate-oxidizing, matrix-associated, flavoprotein subunit (SdhA), an electron transfer iron-sulfur subunit (SdhB) and two hydrophobic membrane anchors (SdhC and SdhD) that provide the binding site for ubiquinone and are required for integration of the complex into the inner mitochondrial membrane. SdhA-D are nucleus-encoded in a wide variety of eukaryotes, including mammals, whereas SdhB-D are specified by the gene-rich mitochondrial genomes of certain protists such as red algae and jakobid flagellates [4]. SdhA invariably appears to be nucleus-encoded.

*Euglena gracilis *is a free-living, flagellated eukaryotic microbe that contains a plastid likely acquired through the engulfment of a green alga [5]. A monophyletic 'Euglenozoa' clade comprising *Euglena *(and related euglenids) along with two aplastidic lineages, the kinetoplastids (encompassing trypanosomatids and bodonids) and the predominantly free-living diplonemids, has been postulated principally on the basis of shared ultrastructural features, including disc-shaped mitochondrial cristae and flagellar paraxonemal rods [6]. Phylogenetic reconstructions based on small subunit ribosomal RNA (SSU rRNA; [7]) and protein [8] sequences established that these physiologically and ecologically disparate taxa likely comprise a (potentially early-branching) monophyletic group. However, the well-documented effects of rapid rates of sequence change, along with the acquisition of a secondary endosymbiont [5] (evidenced by the presence in *Euglena *of a plastid with three surrounding membranes) and possible ephemeral, cryptic endosymbioses [9], have complicated reconstructions of *Euglena*'s evolutionary history. In particular, the transfer of endosymbiont-derived genes to the nucleus has, in effect, yielded a mosaic nuclear genome displaying characteristics of all constituent sources [10]. Moreover, the internal branching patterns within Euglenozoa are still not completely resolved [11], although phylogenies based on conserved protein genes seem to be consistent in placing euglenids at the base of Euglenozoa, with diplonemids and kinetoplastids forming a later diverging sister group [9].

Here we report that in *E. gracilis*, the nucleus-encoded *sdhB *gene is split into two independently transcribed (and presumably independently translated) subgenic modules whose products correspond to the N-terminal and C-terminal halves (referred to here as SdhB-n and SdhB-c, respectively) of a typical SdhB protein. Moreover, in various trypanosome species, we have identified separate genes encoding predicted proteins corresponding to SdhB-n and SdhB-c. The splitting of *sdhB *in *Euglena *and trypanosomatids is an example of a unique molecular character that specifically unites these two phylogenetic groups and raises interesting questions about the evolution and function of euglenozoan SdhB.

## Results and discussion

Relatively few genomic data are available for *Euglena*. Neither nuclear nor mitochondrial genome sequencing projects are currently being undertaken, and only three mitochondrion-encoded protein-coding genes (*cox1*, *cox2 *and *nad6*) have been identified thus far [[12,13], GenBank:AF156178]. Nevertheless, the construction and sequencing of EST libraries generated from mature mRNAs is being exploited to better understand the biochemistry and evolution of this organism. The conserved 24-nucleotide 5' spliced leader (SL) sequence characteristic of *Euglena *nucleus-encoded mRNAs [14] confers a specific advantage in that its presence in an EST confirms that the translated sequence encompasses the complete N-terminus of the corresponding protein. This information is important in predicting the subcellular localization of a given protein product, as the signals required for targeting proteins to various subcellular compartments, including mitochondria, are frequently located at protein N-termini.

**Figure 1:**
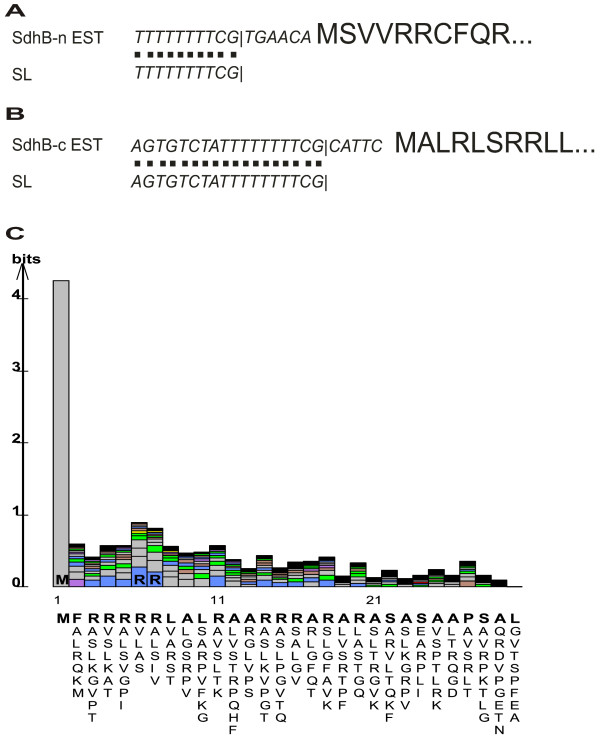
***E. gracilis *consensus EST clusters contain sequences corresponding to spliced leaders**. A. SdhB-n. B. SdhB-c. Letters in italics and smaller font represent nucleic acid sequences, while larger bolded letters represent the N-terminus of the inferred protein sequences. The | character denotes the 3' end of the spliced leader. 'EST' refers to the sequence of the expressed sequence tag and 'SL' refers to the sequence of the spliced leader. C. Consensus mTP profile generated from de-gapped alignment of the N-terminal 30 residues from 107 *E. gracilis *predicted mitochondrion-targeted proteins.

**Figure 2:**
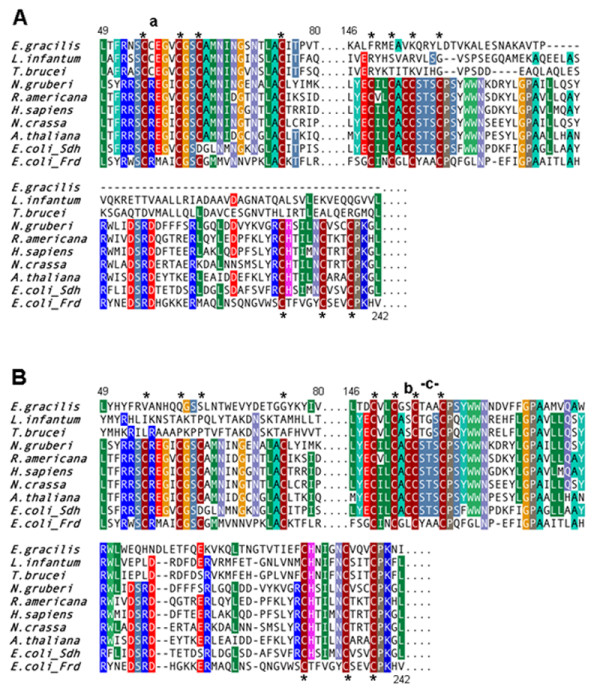
**Alignment of selected regions of *Euglena *and trypanosome SdhB-n and SdhB-c with SdhB and FrdB homologs from other eukaryotes and bacteria**. **A**. SdhB-n. **B**. SdhB-c. Euglenozoan SdhB-n and SdhB-c proteins were aligned with the same set of SdhB and FrdB proteins from other species. Alignments were edited to emphasize regions encompassing conserved Cys residues (denoted by *) responsible for co-ordinating Fe-S clusters. Numbering reflects corresponding amino acid residues in *E. coli *SdhB. Shading of columns indicates amino acid identity of 60% or higher. The letters **a**, **b **and **c **highlight particular residues in the alignment that are discussed in the text. Full organism names and database accession numbers are listed in 'Additional file 4: Accession numbers and allied information'.

Analysis of *Euglena gracilis *EST data demonstrates that SdhB is expressed as separate N- and C-terminal units. The EST clusters from *Euglena *are considered to be complete, as those representing both *sdhB-n *and *sdhB-c *each contains at least 10 of the 3'-most nucleotides ('TTTTTTTTCG') of the conserved SL sequence at the 5' end (Figures 1A,B), an ATG initiation codon a few nucleotides further downstream, and a stop codon near the 3' end of the EST sequence. Moreover, the presence of an SL in *Euglena *ESTs demonstrates that *sdhB-n *and *sdhB-c *are nucleus-encoded in this protist, as mitochondrial transcripts are not known to contain spliced leaders. In total, we identified 8 and 4 ESTs corresponding, respectively, to *Euglena sdhB-n *and *sdhB-c*. Nearly complete *sdhB-n and sdhB-c *ESTs were also found for the related species, *Euglena *(*Astasia*) *longa*. SdhB-n and SdhB-c protein sequences from *E. gracilis *and *E. longa *are 93% and 91% identical, respectively. Although the *E*. *longa *ESTs lack the SL and the sequence corresponding to the extreme N-termini of the two proteins, these ESTs provide further evidence that the *sdhB *modules are transcribed separately in the nucleus of euglenids. Similarly, SdhB sequences inferred from the genome sequences of several trypanosome species indicate that SdhB is also expressed as two separate pieces in these organisms (see Figure 2 for partial protein alignments and additional files 1 and 2 for more extensive alignments). In fact, the two SdhB pieces are encoded on separate chromosomes in the nuclear genomes of trypanosomatids: in *T. brucei*, *sdhB-n *is on chromosome 8 while *sdhB-c *is on chromosome 9, whereas in *L. major*, *sdhB-n *is on chromosome 23 and *sdhB-c *is on chromosome 15. The fragmented nature of *sdhB *in trypanosomatids was evidently not previously noted, as the relevant coding regions in both *T. brucei *and *L. major *are annotated simply as 'succinate dehydrogenase subunits' or 'hypothetical proteins'. BLAST searches did not retrieve any *sdhB *transcripts from the limited diplonemid EST libraries available in TBestDB , including those of *D. papillatum*, *D. ambulator *and *Rhyncopus*; moreover, no mitochondrion-encoded *sdhB *gene was identified during sequencing of the mitochondrial genome of *Diplonema papillatum *[15]. Nevertheless, parsimony considerations argue that SdhB is nucleus-encoded and bipartite in diplonemids as well, given phylogenetic evidence indicating that diplonemids and trypanosomatids [8] or diplonemids and euglenids [7] are sister groups. Exhaustive searches of available genomic and EST data did not turn up evidence of this split SdhB gene arrangement anywhere outside Euglenozoa.

**Figure 3:**
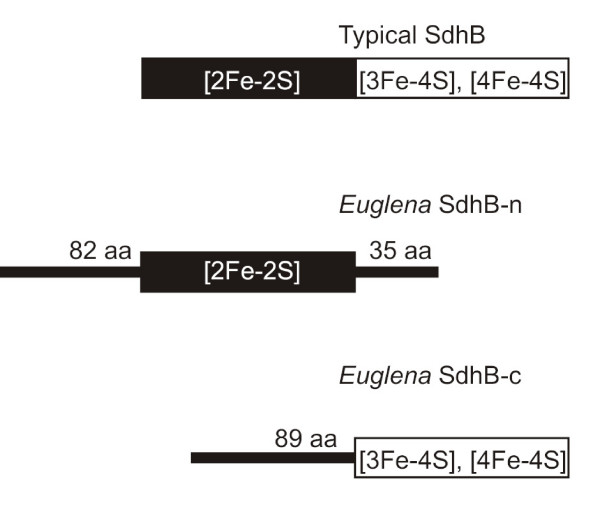
**Organization of protein domains in typical SdhB proteins versus SdhB-n and SdhB-c from *Euglena *and trypanosomatids**. SdhB-n contains the [2Fe-2S]-cluster-binding domain characteristic of the N-terminus of typical SdhB. SdhB-c contains [3Fe-4S]- and [4Fe-4S]-cluster-binding domains that are present in the C-terminal half of SdhB.

Both of the deduced *Euglena *SdhB pieces (Figure 3) are predicted to contain mitochondrial targeting peptides (mTPs). TargetP [16] predicts a mitochondrial localization for SdhB-n and SdhB-c with 91.5% and 96.1% confidence, respectively, whereas the confidence levels with MitoProtII [17] are 99.8% and 95.7%, respectively. Moreover, the N-terminal sequences of SdhB-n and SdhB-c, which are rich in Arg, Ser and hydrophobic residues, closely resemble a consensus *E. gracilis *mTP profile generated from an alignment of 107 predicted mitochondrion-targeted proteins (Figure 1C). That both SdhB-n and SdhB-c contain predicted mTPs is strong evidence that *Euglena *imports both of these separate proteins independently into mitochondria, where they presumably form a heterodimer that effects the role of the classical, covalently continuous SdhB. In trypanosomatids, only SdhB-n is confidently predicted to possess a mTP. The significance of this observation is unclear, although it is possible that SdhB-c is only imported into mitochondria under certain developmental or physiological conditions, or that the protein is imported in a fashion that does not require a cleavable mTP [18].

The discovery of split genes encoding proteins that function within mitochondria is not without precedent. For instance, cytochrome oxidase subunit 2 (Cox2) in the green algae *Chlamydomonas reinhardtii *and *Polytomella *sp. [19] and in several apicomplexan parasites [20] and dinoflagellates [21] is a nucleus-encoded heterodimer specified by two separate subgenic modules. In *Chlamydomonas*, the N-terminal portion of Cox2 has been shown to contain a cleavable N-terminal mTP, whereas the C-terminal unit does not [19]. This situation parallels that reported here for trypanosome SdhB-c, which does not appear to contain a canonical cleavable mTP. In the case of chlamydomonad algae, it has been proposed that a 20-amino acid C-terminal extension in Cox2a (the N-terminal unit) and a 42-amino acid N-terminal extension in Cox2b might facilitate the functional interaction of these two subunits [19]. In the absence of biochemical evidence confirming the length of the mitochondrial targeting peptide, it is not possible to determine unequivocally whether or not trypanosome SdhB-c has an N-terminal extension. On the other hand, SdhB-n from *Euglena *does possess a C-terminal extension of ~35 amino acids, whereas the corresponding trypanosome SdhB-n C-terminal extension is ~105 residues long. Sequence alignments do not indicate any significant similarity between the *Euglena *and trypanosome extensions. As proposed for Cox2 in chlamydomonads, these extensions might allow the dimerization of SdhB-n and SdhB-c in euglenozoans, although bioinformatic analysis does not suggest the presence of obvious protein-protein interaction domains.

From a structural perspective, the split in the SdhB sequence in *Euglena *and trypanosomatids occurs in a region that might be particularly tolerant of such disruption (Figure 3). SdhB contains three iron-sulfur (Fe-S) centers, arranged in a linear chain, that function to transport electrons from SdhA to the membrane-integrated subunits [1]. SdhB from *E. coli *is organized into two domains: an N-terminal domain containing a [2Fe-2S] cluster that forms a fold similar to plant-type ferredoxins and a C-terminal domain that houses the [3Fe-4S] and [4Fe-4S] clusters with a fold similar to bacterial ferredoxins [22]. SdhB-n from *Euglena *contains a predicted Fer2 domain whereas SdhB-c is predicted to contain two Fer4 domains, indicating that the break between *Euglena *SdhB-n and SdhB-c occurs in a region corresponding to the junction between the two *E. coli *domains. Moreover, protein alignments demonstrate that all of the Cys residues required for co-ordination of the three Fe-S clusters in *E. coli *SdhB are accounted for when both SdhB-n and SdhB-c from Euglenozoa are considered. These observations lend further support to the notion that these separate protein halves are functional, as rearrangement occurring within protein domains and/or loss of Fe-S cluster ligands would likely not be tolerated.

Notably, the amino acid sequences of SdhB-n and SdhB-c from Euglenozoa are exceptionally divergent in comparison with SdhB characterized to date in any other organism. In fact, many of the otherwise universally (or nearly universally) conserved residues have been substituted with different ones in Euglenozoa. For instance, a universally conserved Arg (R56 in *E. coli*) is Cys in SdhB-n of both *Euglena *and trypanosomatids (Figure 2A, **a**). Conversely, the conserved Cys corresponding to C154 in *E. coli *is Ser in *Euglena *and trypanosome SdhB-c (Figure 2B, **b**), as well as in SdhB from the unrelated malaria parasite, *Plasmodium falciparum*. The nearby Ser-Thr-Ser motif present in all other SdhB sequences examined here (corresponding to *E. coli *residues 156–158; Figure 2B, **c**) is Thr-Ala-Ala in *Euglena*. Although *E. coli *C154 is not directly responsible for coordinating Fe-S clusters in SdhB, the crystal structure suggests that it contributes a hydrogen bond to the thiol group, important in stabilizing the [4Fe-4S] cluster ligand C152 [1]. It is thought that this H-bond maintains a higher midpoint potential in the [4Fe-4S] cluster. Interestingly, Cheng *et al*. [23] found a direct relationship between the midpoint potential of the [4Fe-4S] cluster and the turnover rates of succinate dehydrogenase, whereas Hudson *et al*. [24] found the inverse for the Fe-S subunit of *E. coli *fumarate reductase (Frd; an homologous enzyme that catalyzes the reduction of fumarate to succinate). Thus, the presence of C154 may favor the *in vivo *oxidation of succinate to fumarate, as opposed to the reverse reaction [23]. *E. coli *FrdB, which has a lower [4Fe-4S] cluster midpoint potential than does *E. coli *SdhB, has a Leu residue instead of the *E. coli *C154 equivalent (Figure 2B, **b**) and a Tyr-Ala-Ala motif (Thr-Ala-Ala in *Euglena*) instead of Ser-Thr-Ser (Figure 2B, **c**). Thus, there exist some interesting parallels between the euglenozoan SdhB and *E. coli *FrdB sequences, although phylogenetic analyses (see additional file 3: SdhB phylogenetic tree) clearly demonstrate that SdhB-n and SdhB-c are SdhB (and not FrdB) homologs. Moreover, it is quite possible that the Ser in euglenozoan SdhB-c contributes a stabilizing hydrogen bond to the [4Fe-4S] cluster (equivalent to the function of C154 of *E. coli*) whereas Leu in FrdB could not. Taken together, the euglenozoan SdhB structure and sequence are intriguing, and emphasize the need for biochemical investigations to fully understand the function and structure of these split proteins.

## Methods

Expressed sequence tags (ESTs) from *E. gracilis *strain Z were prepared as described in [25]. ESTs encoding *Euglena *SdhB were identified by a tBLASTn [26] search of the taxonomically broad EST database (TBestDB; [27]) and GenBank, using SdhB from *Reclinomonas americana *(gi:11466549) as query. Consensus EST sequences specifying SdhB-n and SdhB-c were translated and the inferred protein sequences were subsequently used to query the non-redundant protein sequence database at NCBI (using BLASTp) along with the non-human, non-mouse EST database (est_others) and TBestDB (using tBLASTn). Database accession numbers are given in additional file 4. The programs TargetP [16] and MitoProt II [17] were used to assess the probability of mitochondrial localization for *Euglena *and trypanosome SdhB-n and SdhB-c. When using TargetP for *Euglena *proteins, we selected the 'Plant' organism group in order to include the possibility of plastid-targeting, whereas we selected the 'Animal' organism group for trypanosomatids, as the latter do not contain plastids. MitoProt II contains no option for assessing plastid localization. The consensus *E. gracilis *mTP profile was generated using LogoBar-0.9.12 [28] from a de-gapped alignment of the 30-most N-terminal residues from 107 predicted *E. gracilis *mitochondrion-targeted proteins.

Conserved domains were identified by searching the Pfam and SMART databases at the SMART server [29], using *E. gracilis *SdhB-n and SdhB-c as queries. Protein alignments were constructed using Muscle v3.6 [30] with default parameters and edited with the BioEdit Sequence Alignment Editor. The editing function was used to remove gaps from the non-homologous euglenozoan protein extensions. However, regions corresponding to likely mTPs were left unedited. In the alignment, shading of a given column reflects a minimum of 60% identity.

## List of abbreviations

FAD: flavin adenine dinucleotide (oxidized form); FADH_2_: flavin adenine dinucleotide (reduced form); Fe-S: iron-sulfur; mTP: mitochondrial targeting peptide; SDH: succinate dehydrogenase (succinate-ubiquinone oxidoreductase); SL: spliced leader.

## Competing interests

The authors declare that they have no competing interests.

## Authors' contributions

RMRG discovered the EST and gene sequences corresponding to bipartite SdhB-n and SdhB-c in *Euglena *and trypanosomes and performed bioinformatics analyses. RMRG and MWG prepared the manuscript. Both authors read and approved the final manuscript.

## Supplementary Material

Additional file 1 **Phylogenetically broad alignment of the N-terminal portion of SdhB.** The figure displays more extensive protein alignments of the N-terminal half of SdhB-n than are presented in Figure 2A. This alignment includes SdhB sequences from a phylogenetically broad collection of eukaryotes. Shading of columns represents at least 70% identity. Click here for file

Additional file 2 **Phylogenetically broad alignment of the C-terminal portion of SdhB.** The figure displays more extensive protein alignments of the C-terminal half of SdhB-c than are presented in Figure 2B. See additional file 1 for details. Click here for file

Additional file 3 **Maximum likelihood phylogenetic tree of concatenated SdhB-n and SdhB-c proteins.** This maximum likelihood phylogenetic tree reconstruction demonstrates that the euglenozoan SdhB-n and SdhB-c proteins are orthologs of mitochondrial SdhB (as opposed to FrdB). Euglenozoan SdhB-n and SdhB-c protein sequences were concatenated and aligned with SdhB and FrdB sequences from other eukaryotes and prokaryotes. The alignments were edited and PHYML was used to reconstruct the phylogeny. The WAG amino acid substitution model was used, with no invariable sites, 8 substitution rate categories and an estimated Γ distribution parameter. Nonparametric bootstrap analyses (100) were performed. Click here for file

Additional file 4 **Database accession numbers, annotation, gene location, protein size.** GenBank accession numbers, TBestDB entry information, associated annotations, gene locations (nuclear or mitochondrial DNA), sizes of inferred proteins. Click here for file
