# Supplementary material for: A split and rearranged nuclear gene encoding the iron-sulfur subunit of mitochondrial succinate dehydrogenase in Euglenozoa
Source: BMC Res Notes. 2009 Feb 3;2:16. doi: 10.1186/1756-0500-2-16 (PMC2663770; doi:10.1186/1756-0500-2-16)
Supplement: Additional file 1 — Phylogenetically broad alignment of the N-terminal portion of SdhB. The figure displays more extensive protein alignments of the N-terminal half of SdhB-n than are presented in Figure 2A. This alignment includes SdhB sequences from a phylogenetically broad collection of eukaryotes. Shading of columns represents at least 70% identity. [file 1756-0500-2-16-S1.pdf]

```
E.gracilis      MSVVRRCFQRALRPLGQRAYSLQHARSIVPGVPDTLEVTEGKVTPFKEFSAGR--PSLMTKPQGVKPIEE
E.longa         ------------------------------------MTRPLGAKPI----------------------AE
L.infantum      ------------------------------------MLRKVAPRPYKTMVRRM------------ATTDA
T.cruzi         -------------------------------------MLRKITTPYKISVRRT--------------TST
T.brucei        -------------------------------------MLRKVTSPYKVSIRRT--------------AAS
N.gruberi       -----------------------------------------------------------------MLLSS
R.americana     ----------------------------------------------------------------------
M.californiana  --------------------------MLRSLSTLSRFARSAAPTAEKAAASAK-----------PAAAAM
U.maydis        ------------------------MSLFNVSNGLRTALRPSVASSSRVAAFST--TAAARLATPTSDNVG
N.crassa        ------------------MAALRSSSARVFAAASRPAFRPVVAARGMANLADG----------ATQSQQA
M.ovata         -----------------------------VRTPVTSIVARLASTAATTAAAEP----------ALKTQAA
M.musculus      -----------------------------MAATVGVSLKRGFPAAVLGRVGLQ--------FQACRGAQT
P.parva         -------------------------MLATLSKRATSIVRPGALSAFISTSSEA----------LAAPSKP
C.reinhardtii   ---------------------MLPSLLTNARRGAQAALQPGFLSAFISTTSESLNAAATATASKPAPSRP
A.thaliana      ------------------MASGLIGRLVGTKPSKLATAARLIPARWTSTGAEA-----------ETKASS
B.hominis       ------------------------MFARSLSSLSKVAVRGSVVLPQISAMNFS-----------TGFKIG
G.theta         --------------------------------SVTKAVVGAQASSFHSSASLS-----------LKLQPE
P.tetraurelia   ----------------MLQRLSAVIRRPMFWHGTHHHDSNDATKRLFDTVSSTVKGIQQINYVVEHDPKL
P.falciparum    -----------MLKKYELKGVMNILNKKLCNNKSRNDIIQAYTYIQKRFNNGSINKEFEMKKQVEQINKV
B.natans        ----------------------------MMASLLSTTFSKRLVGGMRAVARNG------------APRPS
R.prowazekii    -----------------------------------MVELRLPSNSVVKKGREH----------------K
E.coli          ----------------------------------------------------------------------

E.gracilis      VPERLPDQAIIQIGRWDPDIN--DFRMDTFVYE--KG-PFMVLDILIAIKAHQDPTLTFRNSCCEGVCGS
E.longa         VPERKPEEAIIQIGRWDPDIN--DFRMDTFVYE--KG-PFMVLDILIAIKAHQDPTLTFRNSCCEGVCGS
L.infantum      AAEPHAKKAVLQLVRFDPETN--SSRVESYEYD-KHH-EYMVLDLLIAVKAHQDPTLAFRSSCCEGVCGS
T.cruzi         APEATSKKAILRLIRFDPTTN--RQRIESYEYD-KQH-EYMVLDLITAVKAHQDPTLAFRASCCEGVCGS
T.brucei        VTAADSKKAILRLIRFDPETN--KQRVESYEYD-KHH-DYMVLDLITAVKAHQDPTLAFRASCCEGVCGS
N.gruberi       FPVDSNALIQYKVFRYDPFIN-SEPWVQLYFISNEAH-SSMLLDNLFYLKNEKDESLSYRRSCREGICGS
R.americana     MNTKKEKIMLFKVYRWNPDKK-EKPHISTYSVDLNSC-GPMVLDALIKIKNEQDSTLTFRRSCREGVCGS
M.californiana  PTKKAPDVRRFQIYRWDPEKN-EKPRLQSYDVDMSQC-GPMVLDVLIKIKNEVDPTLTFRRSCREGICGS
U.maydis        SSGKPQHLKQFKIYRWNPDKPSEKPRLQSYTLDLNQT-GPMVLDALIKIKNEIDPTLTFRRSCREGICGS
N.crassa        SSEQSPKLKTFQIYRWNPDTPTEKPRMQSYTLDLNKT-GPMVLDALVRIKNELDPTLTFRRSCREGICGS
M.ovata         STEKAPRIKTFKIYRWNPEKPNDKPHYDEFKIDLNKC-APMVLDALLKIKNEIDPTLTFRRSCREGVCGS
M.musculus      AAAAAPKIKKFAIYRWDPDKTGDKPRMQTYEVDLNKC-GPMVLDALIKIKNEVDSTLTFRRSCREGICGS
P.parva         AVAKAPLYKEFQIYRWNPDSD-EKPKYASYQVDINNC-GPMMLDVLLKVKDEQDQTLSLRRSCREGICGS
C.reinhardtii   PLAKPPLYKEFQIYRWNPDSD-EKPKYASYQVDINNC-GPMMLDVLLKIKDEQDQTLSLRRSCREGICGS
A.thaliana      GGGRGSNLKTFQIYRWNPDNP-GKPELQNYQIDLKDC-GPMVLDALIKIKNEMDPSLTFRRSCREGICGS
B.hominis       PDGKIPSYKLFSLFRYNPAKD-IKPHYEKYVIDLNDC-GTMVLDALFKIKNEQDTTLAFRRSCREGICGS
G.theta         ANVEGKRIKYFQIYRWDPDQS-SRPTMATYPINLSEC-GPMVLDALLKIKNEQDSTLTFRRSCREGICGS
P.tetraurelia   TVEEKAKMKQFLIYRYDPADENDFPKYVSYYVDLKKI-PPMYLDALLYIKDNYDSSLSLRRSCREGICGS
P.falciparum    NGEVVKRKKKFSIFRYNPTNK-KRPQMETFEVDIDNC-GPMVLDVLIKIKDEIDSTLSFRRSCREGICGS
B.natans        FYRRAMSTKTFEIYRWNPDEG-GEPKMQSYDLDLKEC-GPMVLDALIKIKNEVDPTLTFRRSCREGICGS
R.prowazekii    AQQKMLKPRKVKVYRYDPDLD-ENPTIDSFEIDLSKT-GPMVLDALIKIKNEIDSTLTFRRSCREGICGS
E.coli          ------MRLEFSIYRYNPDVD-DAPRMQDYTLEADEGRDMMLLDALIQLK-EKDPSLSFRRSCREGVCGS

E.gracilis      CAMNINGSNTLACITPV-------TDET-VVFPLTQMPILRDLVVDFRWFFKQVELVQNSTPKPNKALFR
E.longa         CAMNINGSNTLACITPV-------TDET-VVFPLTQMPILRDLVVDFRWFFKQVELVQNSTPKPNKAMFR
L.infantum      CAMNINGINSLACITFA-------QQVT-TVAPLPNFPVIKDFVVDLRHFFQQYAYIRPFVRNANLHRSQ
T.cruzi         CAMNINGINSLACITFS-------QQVT-TVGPLPNFPVIKDFVVDLRHFFRQYAQIRPFVRNTNLDRSR
T.brucei        CAMNINGVNSLACITFS-------QQVT-TVGPLPNFPVIKDFVVDLRHFFRQYAYIRPFVRNVNLDRSR
N.gruberi       CAMNINGENALACLYIMKEHLNVLNNEV-RIFPLPHMPVVKDLIVCMKHFYLQYKSINPFLLKNSISYV-
```

```
R.americana     CAMNIDGTNTLACIKSI----DTNKKEM-KIYPLPHMHIIKDLVPDLSNFYAQYKSIEPWMKTTEKKLD-
M.californiana  CAMNIDGTNTLACLRAV-------DQKSMKIYPLPHMAVIKDLVPDLTNFYRQHASVQPYLKYKTEPAP-
U.maydis        CAMNIDGVNTLACLCRI-----DKQNDT-KIYPLPHMYIVKDLVPDLTQFYKQYRSIEPFLKSNNTPSE-
N.crassa        CAMNINGTNTLACLCRI---PADNSAEM-KIYPLPHTYVVKDLVPDLTLFYKQYKSIKPYLQRDTPSPD-
M.ovata         CAMNIDGENTLACIRPI----DPTTPVS-KILPLPHMYVVKDLVPDLTLFYEQYKSIDPFLRTKGKPTD-
M.musculus      CAMNINGGNTLACTRRI---DTDLSKVS-KIYPLPHMYVIKDLVPDLSNFYAQYKSIEPYLKKKDESQE-
P.parva         CAMNINGTNTLACLCKV-D--RDPGQIT-KVAPLPHMFVVKDLVVDMANFYSQYKSIKPYLQRNQEPAN-
C.reinhardtii   CAMNIDGSNTLACLCKV-N--RDPGHVG-KVAPLPHMFVVKDLVVDMANFYAQYKSIKPYLQKKEAAKG-
A.thaliana      CAMNIDGCNGLACLTKI----QDEASET-TITPLPHMFVIKDLVVDMTNFYNQYKSIEPWLKRKTPASV-
B.hominis       CAMNIDGENGLACLTKI-----VPGSATTTIRPLPHMFVIKDLIPDMTNFYEQYASIKPWLQKKSKVDT-
G.theta         CAMNIDGSNTLACLARI----DTSSSKT-KIYPLPHMYVVKDLVPDMTNFYAQYKTIEPWLQKEGNEGKI
P.tetraurelia   CSMNCNGLHKLACIHAI-D--TDLTQPA-YITPLGHMFVVKDLVVDMTNFYTQYKTIDPYLKRKTPKEG-
P.falciparum    CAMNINGKNGLACLTEV-N--RDKKEIT-EIQPLPNLYVMKDLVPDLTNFYNQYKSIDPWLKRKTKKEK-
B.natans        CAMNIDGGNNLACLSKI----EDNGKPT-KIYPLPHMEVIKDLVPDLNNFYQQYKSIKPWLQPAESSPK-
R.prowazekii    CAMNIDGTNTLACIKPI----EDISGDI-KIYPLPHMKVVKDLVPDMSHFYAQYESIEPWLKNDSPAPS-
E.coli          DGLNMNGKNGLACITPI-SALNQPGKKI-VIRPLPGLPVIRDLVVDMGQFYAQYEKIKPYLLNNGQNPP-

E.gracilis      MEAVKQRYLDTVKALESNAKAVTP----------------------------------------------
E.longa         MEALKQRYLDTVKALDSNAKAVTP----------------------------------------------
L.infantum      VDSIVERYHSVARVLSGVSPSEGQAMEKAQEELASVQKRETTVAALLRIA-----DAAVDAGNATQALSV
T.cruzi         VENIIERYDTLRKVIHGVSPHGIPTDSDVPRGGNVETEVVGLLRLLDAIS---------EAGNVTHLVST
T.brucei        LDNIVERYKTITKVIHGVPSDD---------EAQLAQLESKSGAQTDVMALLQLLDAVCESGNVTHLIRT
N.gruberi       -------SSYLTTKLLTLGFNTSMLLTLPKKENIQFDRYLLNGL---YECILCACCSTSCPSYWWNKDRY
R.americana     -------KEFYQSRN----------------------DREKLDGL---YECVLCACCSTSCPSYWWNSDKY
M.californiana  ------GVSNLQSEE----------------------QRAKLNGL---YECVLCACCSTSCPSYWWNGDKY
U.maydis        -------GEHLQSPE----------------------ERRRLDGL---YECILCACCSTSCPSYWWNQDEY
N.crassa        ------GKEYRQSKA----------------------DRKKLDGL---YECILCACCSTSCPSYWWNSEEY
M.ovata         ------GKEFKQTIT----------------------DRKKLDGM---YECVLCACCSTSCPSYWWNSDKY
M.musculus      -----GKQQYLQSIE----------------------DREKLDGL---YECILCACCSTSCPSYWWNGDKY
P.parva         ------GSEYYQSKE----------------------DRLKLDGM---YECILCACCSTSCPSYWWNSDKY
C.reinhardtii   -------QEFYQSKE----------------------SRAKLDGL---YECILCACCSTSCPSYWWNSDKY
A.thaliana      -----PAKEILQSKK----------------------DRAKLDGM---YECILCACCSTSCPSYWWNPESY
B.hominis       ----NYECENLQSYE----------------------DRQKLDGL---YECILCACCSTSCPSYWWHPDKY
G.theta         KDDNGNFKENYMSKK----------------------DRDVLDGM---YECILCACCSTSCPSYWWNSEKY
P.tetraurelia   ------NKEYIQSVE----------------------DRKLLDGL---YECVLCACCSTSCPSYWWHPDRY
P.falciparum    -----GQKEFYQSIE----------------------DRKKLDGL---YECIMCASCSTSCPSYWWNPEYY
B.natans        ----AQDGEYLQTKE----------------------DRKLLDGM---YECILCACCSTSCPSYWWNGDKY
R.prowazekii    ------NSERLQSIK----------------------DREKLDGL---YECILCACCSTSCPSYWWNGDKY
E.coli          ------AREHLQMPE----------------------QREKLDGL---YECILCACCSTSCPSFWWNPDKF

E.gracilis      ----------------------------------------------------------------------
E.longa         ----------------------------------------------------------------------
L.infantum      LEKVEQQGVVLDSTKVTEMIERALKNFAATSK--------------------------------------
T.cruzi         LEELEAKGVQLDQAKVKELIETTLRKHGERTK--------------------------------------
T.brucei        LEALQERGMQLDQGKVKALIEETLQNYKKRKLGA------------------------------------
N.gruberi       LGPAILLQSYRWLIDSRDDFFFSRLGQLD----DVYKVGRCHSILNCVSCCPKGLNPAEAINNIKLLINL
R.americana     LGPAVLLQAYRWIVDSRDQGTRERLQYLE----DPFKLYRCHTILNCTKTCPKHLNPAQAIAKIKQNITL
M.californiana  LGPAILLXAYRWIADSRDQSTRERLESLD----DAFKLYRCHTIMNCSRT--------------------
U.maydis        LGPAVLMQAYRWMADSRDDFGEERRQKLE----NTFSLYRCLTIMNCSRTCPKNLNPGKAIAQIKKDMAV
N.crassa        LGPAILLQSYRWLADSRDERTAERKDALN----NSMSLYRCHTILNCTRTCPKGLNPGLAIANIKKELAF
M.ovata         LGPAALMQAYRWVADTRDEFTKERLEKLK----DPFSVYRCHTIMNCAKTCPKHLNPGKAIAELKKKLAT
M.musculus      LGPAVLMQAYRWMIDSRDDFTEERLAKLQ----DPFSVYRCHTIMNCTQTCPKGLNPGKAIAEIKKMMAT
```

```
P.parva          LGPSVLLAAYRWIIDSRDDATKERIAQLD----DAFKLFRCKTIMNCATVCPKGLNPGKAIAKLKQSVAR
C.reinhardtii    LGPAVLLAAYRWIIDSRDDMTSERMKEVD----DAYKLYRCKTIMNCATVCPKGLNPGKAINKIKQSLAK
A.thaliana       LGPAALLHANRWISDSRDEYTKERLEAID----DEFKLYRCHTILNCARACPKGLNPGKQITHIKQLQR-
B.hominis        LGPSILQQAYRWIADSRDEMTEERLKDLD----DTYKLYRCHAIMNCTHACPKNLNPGRSIHKIKHALHH
G.theta          LGPAVLMQAYRWICDSRDQNTKKRMEMLD----DTYKLYRCHTIMNCTKTCPKSLNPGLAIARLKRKLVT
P.tetraurelia    LGPAVLMQAYRWIVDSRDEYTDERLEKLA----EDVKVEDCQNIGMCSFTCPKGLDPQRSMNHLMKLIEE
P.falciparum     LGPATLMQAYRWIVDSRDEYTKERLMEVN----DTMKLYRCHGIMNCTMCCPKGLDPAKAIKDMKNLVQE
B.natans         LGPAVLMQAYRWIADSRDGDTQARLENLD----DSFKLYRCKTIFNCTNTCPKGLNPGRAVQQIKKAIHD
R.prowazekii     LGPAILLQAYRWIADSRDDNTGARLEALE----DPFKLYRCHTIMNCTKTCPKGLNPAKAIGRVKNLIAE
E.coli           IGPAGLLAAYRFLIDSRDTETDSRLDGLS----DAFSVFRCHSIMNCVSVCPKGLNPTRAIGHIKSMLLQ

E.gracilis       ----------------------
E.longa          ----------------------
L.infantum       ----------------------
T.cruzi          ----------------------
T.brucei         ----------------------
N.gruberi        NTK-------------------
R.americana      LT--------------------
M.californiana   ----------------------
U.maydis         GAPKASERPIMASS--------
N.crassa         ----------------------
M.ovata          V---------------------
M.musculus       YKEKRALA--------------
P.parva          GSAV------------------
C.reinhardtii    GSPV------------------
A.thaliana       ----------------------
B.hominis        MH--------------------
G.theta          G---------------------
P.tetraurelia    YKERKIASATL-----------
P.falciparum     NFSEDTIKEHSQYIKSKMEKTK
B.natans         GEH-------------------
R.prowazekii     RHGV------------------
E.coli           RNA-------------------
```
