# Supplementary material for: A split and rearranged nuclear gene encoding the iron-sulfur subunit of mitochondrial succinate dehydrogenase in Euglenozoa
Source: BMC Res Notes. 2009 Feb 3;2:16. doi: 10.1186/1756-0500-2-16 (PMC2663770; doi:10.1186/1756-0500-2-16)
Supplement: Additional file 2 — Phylogenetically broad alignment of the C-terminal portion of SdhB. The figure displays more extensive protein alignments of the C-terminal half of SdhB-c than are presented in Figure 2B. See additional file 1 for details. [file 1756-0500-2-16-S2.pdf]

```
E.gracilis      ----------------------------------------------------------------------
E.longa         ----------------------------------------------------------------------
L.infantum      ----------------------------------------------------------------------
T.cruzi         ----------------------------------------------------------------------
T.brucei        ----------------------------------------------------------------------
N.gruberi       ------------------------------------------------------MLLSSFPVDSNALIQY
R.americana     -----------------------------------------------------------MNTKKEKIMLF
M.californiana  ---------------MLRSLSTLSRFARSAAPTAEKAAASAK-----------PAAAAMPTKKAPDVRRF
U.maydis        -------------MSLFNVSNGLRTALRPSVASSSRVAAFST--TAAARLATPTSDNVGSSGKPQHLKQF
N.crassa        -------MAALRSSSARVFAAASRPAFRPVVAARGMANLADG----------ATQSQQASSEQSPKLKTF
M.ovata         -----------------VRTPVTSIVARLASTAATTAAAEPA-----------LKTQAASTEKAPRIKTF
M.musculus      ---------------MAATVGVSLKRGFPAAVLGRVGLQFQA-----------CRGAQTAAAAAPKIKKF
P.parva         --------------MLATLSKRATSIVRPGALSAFISTSSEA----------LAAPSKPAVAKAPLYKEF
C.reinhardtii   ----------MLPSLLTNARRGAQAALQPGFLSAFISTTSESLNAAATATASKPAPSRPPLAKPPLYKEF
A.thaliana      -------MASGLIGRLVGTKPSKLATAARLIPARWTSTGAEA-----------ETKASSGGGRGSNLKTF
B.hominis       -------------MFARSLSSLSKVAVRGSVVLPQISAMNFS-----------TGFKIGPDGKIPSYKLF
G.theta         ---------------------SVTKAVVGAQASSFHSSASLS-----------LKLQPEANVEGKRIKYF
P.tetraurelia   -----MLQRLSAVIRRPMFWHGTHHHDSNDATKRLFDTVSSTVKGIQQINYVVEHDPKLTVEEKAKMKQF
P.falciparum    MLKKYELKGVMNILNKKLCNNKSRNDIIQAYTYIQKRFNNGSINKEFEMKKQVEQINKVNGEVVKRKKKF
B.natans        ------------------MMASLLSTTFSKRLVGGMRAVARN-----------GAPRPSFYRRAMSTKTF
R.prowazekii    -----------------------------MVELRLPSNSVVK-----------KGREHKAQQKMLKPRKV
E.coli          -----------------------------------------------------------------MRLEF

E.gracilis      ----------------------------------------------------------------------
E.longa         ----------------------------------------------------------------------
L.infantum      ----------------------------------------------------------------------
T.cruzi         ----------------------------------------------------------------------
T.brucei        ----------------------------------------------------------------------
N.gruberi       KVFRYDPFIN-SEPWVQLYFISNEAHSSMLL-DNLFYLKNEKDESLSYRRSCREGICGSCAMNINGENAL
R.americana     KVYRWNPDKK-EKPHISTYSVDLNSCGPMVL-DALIKIKNEQDSTLTFRRSCREGVCGSCAMNIDGTNTL
M.californiana  QIYRWDPEKN-EKPRLQSYDVDMSQCGPMVL-DVLIKIKNEVDPTLTFRRSCREGICGSCAMNIDGTNTL
U.maydis        KIYRWNPDKPSEKPRLQSYTLDLNQTGPMVL-DALIKIKNEIDPTLTFRRSCREGICGSCAMNIDGVNTL
N.crassa        QIYRWNPDTPTEKPRMQSYTLDLNKTGPMVL-DALVRIKNELDPTLTFRRSCREGICGSCAMNINGTNTL
M.ovata         KIYRWNPEKPNDKPHYDEFKIDLNKCAPMVL-DALLKIKNEIDPTLTFRRSCREGVCGSCAMNIDGENTL
M.musculus      AIYRWDPDKTGDKPRMQTYEVDLNKCGPMVL-DALIKIKNEVDSTLTFRRSCREGICGSCAMNINGGNTL
P.parva         QIYRWNPDSD-EKPKYASYQVDINNCGPMML-DVLLKVKDEQDQTLSLRRSCREGICGSCAMNINGTNTL
C.reinhardtii   QIYRWNPDSD-EKPKYASYQVDINNCGPMML-DVLLKIKDEQDQTLSLRRSCREGICGSCAMNIDGSNTL
A.thaliana      QIYRWNPDNP-GKPELQNYQIDLKDCGPMVL-DALIKIKNEMDPSLTFRRSCREGICGSCAMNIDGCNGL
B.hominis       SLFRYNPAKD-IKPHYEKYVIDLNDCGTMVL-DALFKIKNEQDTTLAFRRSCREGICGSCAMNIDGENGL
G.theta         QIYRWDPDQS-SRPTMATYPINLSECGPMVL-DALLKIKNEQDSTLTFRRSCREGICGSCAMNIDGSNTL
P.tetraurelia   LIYRYDPADENDFPKYVSYYVDLKKIPPMYL-DALLYIKDNYDSSLSLRRSCREGICGSCSMNCNGLHKL
P.falciparum    SIFRYNPTNK-KRPQMETFEVDIDNCGPMVL-DVLIKIKDEIDSTLSFRRSCREGICGSCAMNINGKNGL
B.natans        EIYRWNPDEG-GEPKMQSYDLDLKECGPMVL-DALIKIKNEVDPTLTFRRSCREGICGSCAMNIDGGNNL
R.prowazekii    KVYRYDPDLD-ENPTIDSFEIDLSKTGPMVL-DALIKIKNEIDSTLTFRRSCREGICGSCAMNIDGTNTL
E.coli          SIYRYNPDVD-DAPRMQDYTLEADEGRDMMLLDALIQLK-EKDPSLSFRRSCREGVCGSDGLNMNGKNGL

E.gracilis      ------------MALRLSRRLLSANPKHHSKLYHYFRVANHQQGSSLNTWEVYDETGGYKYIVDLKVPSS
E.longa         --------------------------------YHYFRVANHLQGSSFNTWEVYDETGGYKYLVDLKVYSS
L.infantum      ---------------------MPSAPLPGQLANYSSPLYMYRHLIKNSTAKTPQLYTAKDNSKTAMHLL-
T.cruzi         ---------------------MPSAPLTGEVARYSSPLFMYRRIIKNAAPKPETVFTAKDNSKTAFHVLT
T.brucei        ---------------------MPAAPLPGEVAKFCSPLYMHKRILRAAAPKPPTVFTAKDNSKTAFHVVT
N.gruberi       ACLYIMKEHLNVLNNEVRIFPLPHMPVVKDLIVCMKHFYLQYKSINPFLLKNSISYV--------SSYLT
```

```
R.americana      ACIKSI-D--TNKKEM-KIYPLPHMHIIKDLVPDLSNFYAQYKSIEPWMKTTEKKLD--------KEFYQ
M.californiana   ACLRAV-D--QKSM---KIYPLPHMAVIKDLVPDLTNFYRQHASVQPYLKYKTEPAP-------GVSNLQ
U.maydis         ACLCRI-D--KQNDT--KIYPLPHMYIVKDLVPDLTQFYKQYRSIEPFLKSNNTPSE--------GEHLQ
N.crassa         ACLCRI-P--ADNSAEMKIYPLPHTYVVKDLVPDLTLFYKQYKSIKPYLQRDTPSPD-------GKEYRQ
M.ovata          ACIRPI-D--PTTPVS-KILPLPHMYVVKDLVPDLTLFYEQYKSIDPFLRTKGKPTD-------GKEFKQ
M.musculus       ACTRRI-D--TDLSKVSKIYPLPHMYVIKDLVPDLSNFYAQYKSIEPYLKKKDESQE------GKQQYLQ
P.parva          ACLCKV-D--RDPGQITKVAPLPHMFVVKDLVVDMANFYSQYKSIKPYLQRNQEPAN-------GSEYYQ
C.reinhardtii    ACLCKV-N--RDPGHVGKVAPLPHMFVVKDLVVDMANFYAQYKSIKPYLQKKEAAKG--------QEFYQ
A.thaliana       ACLTKI-Q--DEASET-TITPLPHMFVIKDLVVDMTNFYNQYKSIEPWLKRKTPASV------PAKEILQ
B.hominis        ACLTKI-V--PGSATT-TIRPLPHMFVIKDLIPDMTNFYEQYASIKPWLQKKSKVDT-----NYECENLQ
G.theta          ACLARI-D--TSSSKT-KIYPLPHMYVVKDLVPDMTNFYAQYKTIEPWLQKEGNEGKIKDDNGNFKENYM
P.tetraurelia    ACIHAI-D--TDLTQPAYITPLGHMFVVKDLVVDMTNFYTQYKTIDPYLKRKTPKEG-------NKEYIQ
P.falciparum     ACLTEV-N--RDKKEITEIQPLPNLYVMKDLVPDLTNFYNQYKSIDPWLKRKTKKEK------GQKEFYQ
B.natans         ACLSKI-E--DNGKPT-KIYPLPHMEVIKDLVPDLNNFYQQYKSIKPWLQPAESSPK-----AQDGEYLQ
R.prowazekii     ACIKPI-E--DISGDI-KIYPLPHMKVVKDLVPDMSHFYAQYESIEPWLKNDSPAPS-------NSERLQ
E.coli           ACITPI-SALNQPGKKIVIRPLPGLPVIRDLVVDMGQFYAQYEKIKPYLLNNGQNPP-------AREHLQ

E.gracilis       NGNTATKAQPAIVQWHGFKMTHPSVVAQFMDLTDCVLCGSCTAACPSYWWNNDVFFGPAAMVQAWRWLWE
E.longa          TNNVTSKSQPAIVQWHGFKMTHPSVVAQFMELTDCVLCGSCTAACPQYWWNNDVFFGPAAMVQAWRWLWE
L.infantum       -TRRAANANYTVNRWYLKEKVDSSNRMRLEGLYECVLCASCTGSCPQYWWNREHFLGPAVLLQSYRWLVE
T.cruzi          QRPGFGNNPYAVSRWYLKEKVHSSNRNRLEGLYECVLCASCTGSCPQYWWNREQFLGPAVLLQSYRWLIE
T.brucei         QRPGFATTPYTVSRWYLKEKVHSSNRNRLEGLYECVLCASCTGSCPQYWWNRELFLGPAVLLQSYRWLIE
N.gruberi        TKLLTLGFNTSMLLTLPKKENIQFDRYLLNGLYECILCACCSTSCPSYWWNKDRYLGPAILLQSYRWLID
R.americana      SRN---------------------DREKLDGLYECVLCACCSTSCPSYWWNSDKYLGPAVLLQAYRWIVD
M.californiana   SEE---------------------QRAKLNGLYECVLCACCSTSCPSYWWNGDKYLGPAILLXAYRWIAD
U.maydis         SPE---------------------ERRRLDGLYECILCACCSTSCPSYWWNQDEYLGPAVLMQAYRWMAD
N.crassa         SKA---------------------DRKKLDGLYECILCACCSTSCPSYWWNSEEYLGPAILLQSYRWLAD
M.ovata          TIT---------------------DRKKLDGMYECVLCACCSTSCPSYWWNSDKYLGPAALMQAYRWVAD
M.musculus       SIE---------------------DREKLDGLYECILCACCSTSCPSYWWNGDKYLGPAVLMQAYRWMID
P.parva          SKE---------------------DRLKLDGMYECILCACCSTSCPSYWWNSDKYLGPSVLLAAYRWIID
C.reinhardtii    SKE---------------------SRAKLDGLYECILCACCSTSCPSYWWNSDKYLGPAVLLAAYRWIID
A.thaliana       SKK---------------------DRAKLDGMYECILCACCSTSCPSYWWNPESYLGPAALLHANRWISD
B.hominis        SYE---------------------DRQKLDGLYECILCACCSTSCPSYWWHPDKYLGPSILQQAYRWIAD
G.theta          SKK---------------------DRDVLDGMYECILCACCSTSCPSYWWNSEKYLGPAVLMQAYRWICD
P.tetraurelia    SVE---------------------DRKLLDGLYECVLCACCSTSCPSYWWHPDRYLGPAVLMQAYRWIVD
P.falciparum     SIE---------------------DRKKLDGLYECIMCASCSTSCPSYWWNPEYYLGPATLMQAYRWIVD
B.natans         TKE---------------------DRKLLDGMYECILCACCSTSCPSYWWNGDKYLGPAVLMQAYRWIAD
R.prowazekii     SIK---------------------DREKLDGLYECILCACCSTSCPSYWWNGDKYLGPAILLQAYRWIAD
E.coli           MPE---------------------QREKLDGLYECILCACCSTSCPSFWWNPDKFIGPAGLLAAYRFLID

E.gracilis       QHNDLETFQEKVKQLTNGTVTIEFCHNIGNCVQVCPKNIQVDRVMNSIRMLASLN---------------
E.longa          QHNDLETFQEKVKQLTNGTVSIEFCHNIGNCVQVCPKNIQVDQVMNSIRMLASLN---------------
L.infantum       PLDRD--FDERVRMFETG-NLVNMCHNIFNCSITCPKFLNPGLASKEIKRLSSPSAKRVGPAIEMEPVTS
T.cruzi          PLDRD--YDSRVKMFEHG-PLVNFCHNIYNCSITCPKFLNPAYASKEIKRMSSPVTPRVPPPLDAKIIKE
T.brucei         PLDRD--FDSRVKMFEHG-PLVNFCHNIFNCSITCPKFLNPGMASKEIKRLSSPATLRVGPPLEDAKVAS
N.gruberi        SRDDF--FFSRLGQLDDV-YKVGRCHSILNCVSCCPKGLNPAEAINNIKLLINLNTK-------------
R.americana      SRDQG--TRERLQYLEDP-FKLYRCHTILNCTKTCPKHLNPAQAIAKIKQNITLLT--------------
M.californiana   SRDQS--TRERLESLDDA-FKLYRCHTIMNCSRT-------------------------------------
U.maydis         SRDDF--GEERRQKLENT-FSLYRCLTIMNCSRTCPKNLNPGKAIAQIKKDMAVGAPKASERPIMASS--
N.crassa         SRDER--TAERKDALNNS-MSLYRCHTILNCTRTCPKGLNPGLAIANIKKELAF----------------
M.ovata          TRDEF--TKERLEKLKDP-FSVYRCHTIMNCAKTCPKHLNPGKAIAELKKKLATV---------------
M.musculus       SRDDF--TEERLAKLQDP-FSVYRCHTIMNCTQTCPKGLNPGKAIAEIKKMMATYKEKRALA---------
```

```
P.parva          SRDDA--TKERIAQLDDA-FKLFRCKTIMNCATVCPKGLNPGKAIAKLKQSVARGSAV------------
C.reinhardtii    SRDDM--TSERMKEVDDA-YKLYRCKTIMNCATVCPKGLNPGKAINKIKQSLAKGSPV------------
A.thaliana       SRDEY--TKERLEAIDDE-FKLYRCHTILNCARACPKGLNPGKQITHIKQLQR----------------
B.hominis        SRDEM--TEERLKDLDDT-YKLYRCHAIMNCTHACPKNLNPGRSIHKIKHALHHMH-------------
G.theta          SRDQN--TKKRMEMLDDT-YKLYRCHTIMNCTKTCPKSLNPGLAIARLKRKLVTG--------------
P.tetraurelia    SRDEY--TDERLEKLAED-VKVEDCQNIGMCSFTCPKGLDPQRSMNHLMKLIEEYKERKIASATL-----
P.falciparum     SRDEY--TKERLMEVNDT-MKLYRCHGIMNCTMCCPKGLDPAKAIKDMKNLVQENFSEDTIKEHSQYIKS
B.natans         SRDGD--TQARLENLDDS-FKLYRCKTIFNCTNTCPKGLNPGRAVQQIKKAIHDGEH------------
R.prowazekii     SRDDN--TGARLEALEDP-FKLYRCHTIMNCTKTCPKGLNPAKAIGRVKNLIAERHGV-----------
E.coli           SRDTE--TDSRLDGLSDA-FSVFRCHSIMNCVSVCPKGLNPTRAIGHIKSMLLQRNA------------

E.gracilis       -------------
E.longa          -------------
L.infantum       GKEAGKRSLAQMQ
T.cruzi          -------------
T.brucei         KY-----------
N.gruberi        -------------
R.americana      -------------
M.californiana   -------------
U.maydis         -------------
N.crassa         -------------
M.ovata          -------------
M.musculus       -------------
P.parva          -------------
C.reinhardtii    -------------
A.thaliana       -------------
B.hominis        -------------
G.theta          -------------
P.tetraurelia    -------------
P.falciparum     KMEKTK-------
B.natans         -------------
R.prowazekii     -------------
E.coli           -------------
```
