# Supplementary figures and images for: A split and rearranged nuclear gene encoding the iron-sulfur subunit of mitochondrial succinate dehydrogenase in Euglenozoa
Source: BMC Res Notes. 2009 Feb 3;2:16. doi: 10.1186/1756-0500-2-16 (PMC2663770; doi:10.1186/1756-0500-2-16)

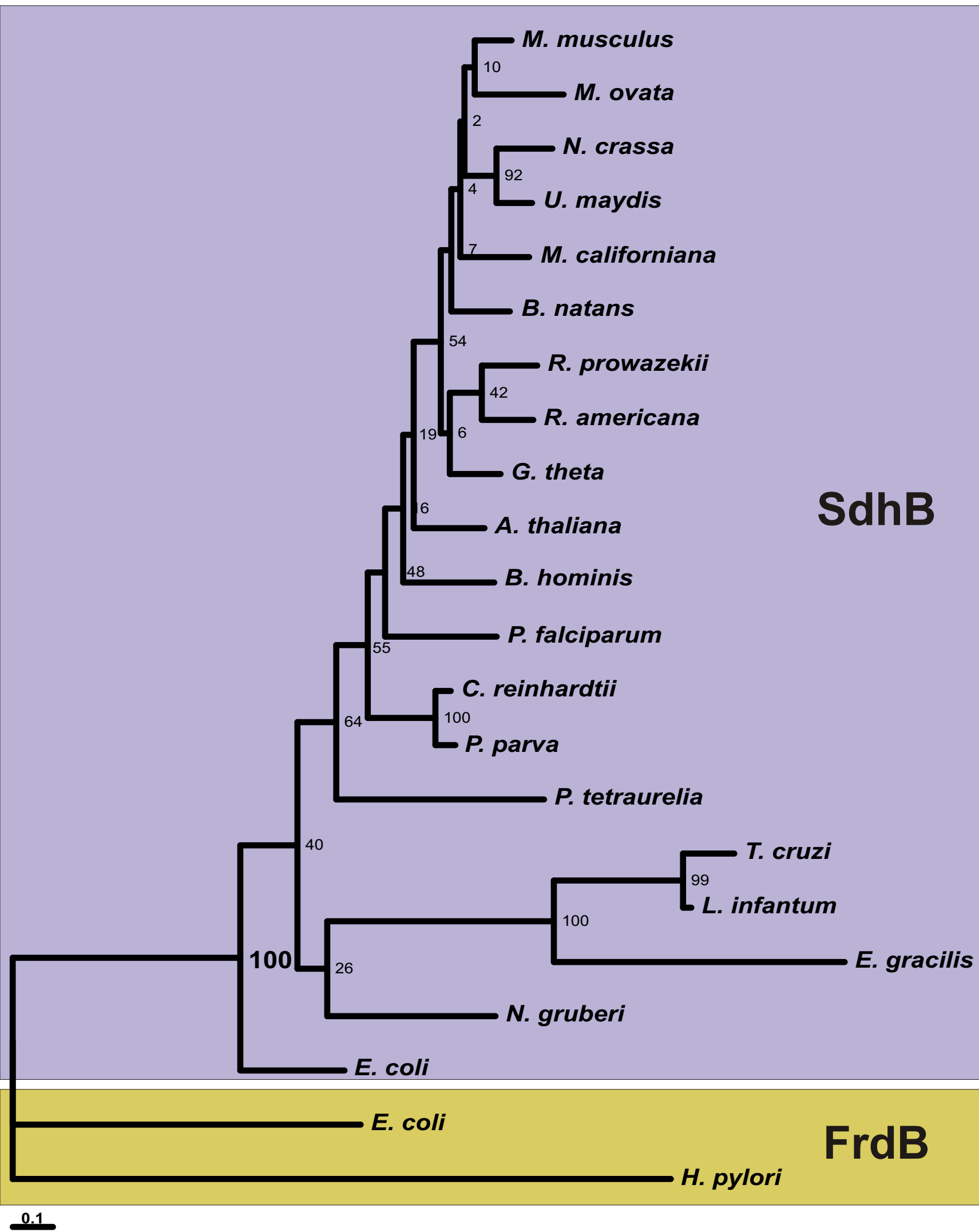

Supplement: Additional file 3 — Maximum likelihood phylogenetic tree of concatenated SdhB-n and SdhB-c proteins. This maximum likelihood phylogenetic tree reconstruction demonstrates that the euglenozoan SdhB-n and SdhB-c proteins are orthologs of mitochondrial SdhB (as opposed to FrdB). Euglenozoan SdhB-n and SdhB-c protein sequences were concatenated and aligned with SdhB and FrdB sequences from other eukaryotes and prokaryotes. The alignments were edited and PHYML was used to reconstruct the phylogeny. The WAG amino acid substitution model was used, with no invariable sites, 8 substitution rate categories and an estimated Γ distribution parameter. Nonparametric bootstrap analyses (100) were performed. [file 1756-0500-2-16-S3.pdf]
